# Utero-cutaneous fistula following cesarean section: A case report

**DOI:** 10.1016/j.radcr.2021.09.069

**Published:** 2021-11-01

**Authors:** Abdelhamid Jadib, Houria Tabakh, Lamiaa Chahidi El Ouazzani, Othmane Kardi, Abdellatif Siwane, Najwa Touil, Omar Kacimi, Nabil Chikhaoui

**Affiliations:** Emergency Radiology Division, Faculty of Medicine and Pharmacy of Casablanca, Ibn Rochd University Hospital, 1, quartiers des hôpitaux, Casablanca, Morocco, 20100

**Keywords:** Utero-cutaneous fistula, Cesarian section, Imaging

## Abstract

Utero-cutaneous fistula is a rare pathology. It mostly occurs consecutively to surgical intervention such as Cesarean section. Blood discharge from the cesarean scar during menstruation is a quasi-pathognomonic feature. Imaging modalities, particularly with the injection of contrast material through the cutaneous fistulous opening, confirm the diagnosis. The management is mainly surgical.

We report the case of a utero-cutaneous fistula in a 27-year-old lady, with systemic lupus erythematosus. She presented seven months after her third cesarean section with pain and blood discharge from the cutaneous scar during menstruation for four months. A pelvic CT scan with the injection of the contrast material through the cutaneous fistulous opening confirmed the diagnosis of utero-cutaneous fistula. Surgical management was successful.

## Key points

Utero-cutaneous fistula is a rare condition that manifests with cyclic pain and blood discharge of the abdominal wall.

The confirmation of the diagnosis is based on imaging with opacification of the fistulous tract.

The primary treatment is surgical excision of the fistulous tract.

## Introduction

Utero-cutaneous fistula is an extremely rare condition and its pathophysiology is not fully understood. It mostly occurs consecutively to surgical intervention such as Cesarean section. Blood discharge from the cutaneous fistulous opening during the menstrual bleeding is almost pathognomonic [1].

We report the case of a 27-year-old woman, with systemic lupus erythematosus. The diagnosis of the utero-cutaneous fistula was confirmed by imaging. The surgical management was successful and consisted of the total excision of the fistulous tract, without hysterectomy.

## Case presentation

A 27-year-old woman, with systemic lupus erythematosus since 2014, under long-term corticosteroid therapy and Hydroxychloroquine. She presented pain and cyclic blood discharge for 4 months from her third Cesarean section that was performed 7 months ago.

**Fig. 1:**
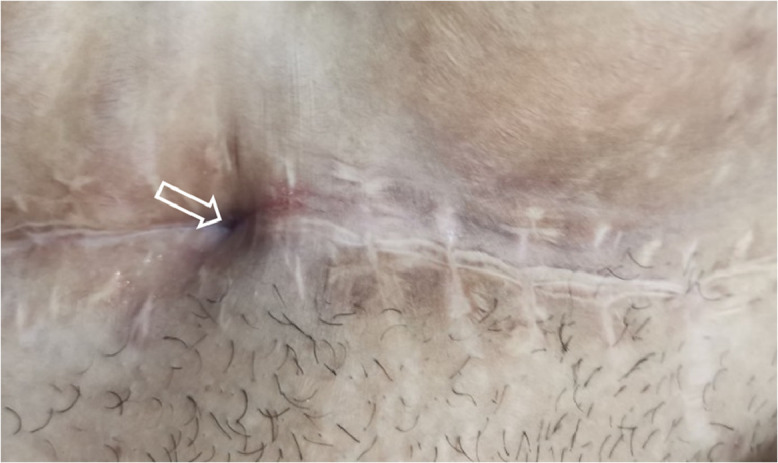
A clinical image of the cesarean section scar with skin retraction and fistulous opening at its right end (arrow).

Examination of the abdominal wall revealed a cutaneous retraction and a fistulous opening at the right end of the scar, without a palpable nodule (Fig. 1).

**Fig. 2:**
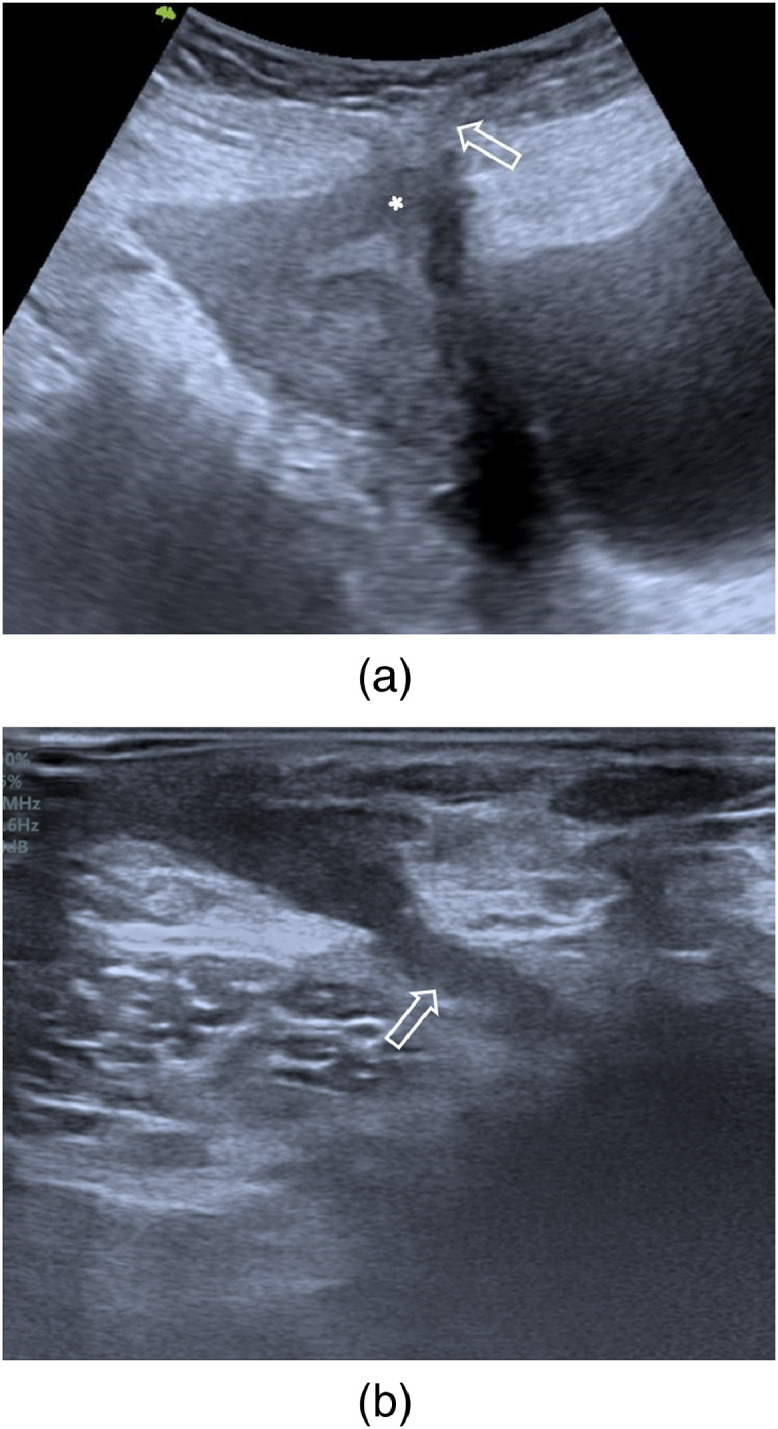
Transabdominal ultrasound with a low-frequency transducer in the sagittal plane (a) and a high-frequency transducer in the axial plane (b) showing a fistulous tract extending from the anterior body of the uterus, that shows a focal myometrial thinning related to the cesarean section scar (asterisk), to the right end of the cutaneous scar (arrow).

A pelvic transabdominal ultrasound was performed, showing a fistulous tract extending from the anterior body of the uterus, that shows a focal myometrial thinning related to the cesarean section scar, to the right end of the cutaneous scar. It was approximately 2 mm in diameter, 31 mm in length (Fig. 2).

**Fig. 3:**
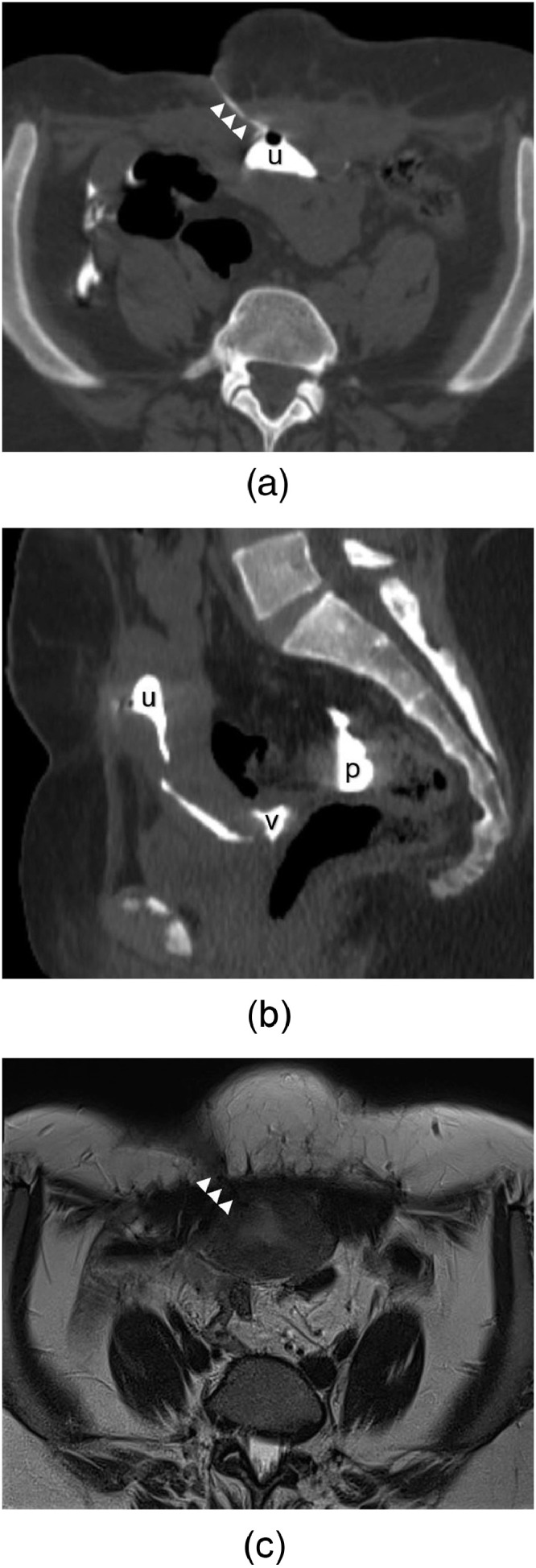
Pelvic CT scan with an injection of diluted iodinated contrast medium through the cutaneous fistulous opening, in the axial (a) and the sagittal (b) planes, showing the opacification of the fistulous tract (arrows) and the uterine cavity (u), with spillage of the contrast medium into the peritoneal cavity through the fallopian tubes (p) and into the vagina through the cervix (v). On pelvic MRI axial T2-weighted sequence (c), the fistulous tract shows a high signal intensity (arrows).

A pelvic computed tomography (CT) was performed with injection of diluted iodinated contrast medium through the cutaneous fistulous opening, showing the opacification of the fistulous tract described on ultrasound, as well as the uterine cavity, confirming the diagnostic diagnosis (Fig. 3).

Pelvic magnetic resonance imaging (MRI) was performed. The fistulous tract showed low signal intensity on T1-weighted sequences, low signal intensity on T2-weighted sequences, high signal intensity on T2-weighted sequences with fat saturation, and enhanced after intravenous Gadolinium contrast medium administration (Fig. 3).

The surgical management was successful. It consists of total excision of the fistulous tract with uterus conservation. It leads to the disappearance of cyclic blood discharge and pain.

The histopathological examination was compatible with parietal endometriosis.

## Discussion

Utero-cutaneous fistula is an extremely rare condition, with few cases being reported in the literature. It is an abnormal communication between the cutaneous surface and the uterine cavity. The main manifestation is blood discharge from the cutaneous fistulous opening during the menstrual period, which is almost pathognomonic [1].

The pathophysiology is not well understood. Several possible causes may predispose to this condition. Most of the reported cases were consecutive to Cesarean sections. Others were causes reported such as multiple surgeries in the abdomen, use of drains, incomplete closure of incisions, septic abortion, pelvic abscesses, incomplete placenta removal, uterovaginal malformation, pelvic actinomycosis due to intrauterine devices, curettage, difficult delivery, or use of forceps [2].

Multiple imaging techniques confirming the diagnosis had been reported. Fistulography with the injection of the contrast material through the cutaneous fistulous opening confirms the connection to the uterus. Hysterosalpingography with a methylene blue injection through the cervix is used when the cutaneous opening is too small. CT scan with intravenous injection of contrast medium and MRI can also identify the fistula. Hysteroscopy allows direct visualization of the internal fistulous opening [2].

The primary treatment of utero-cutaneous fistula is surgical excision of the fistulous tract without or with a subtotal or total hysterectomy [3].

Gonadotropin-releasing hormone agonists would induce amenorrhea secondary to endometrial atrophy, resulting in contracture and healing of the fistulous tract. It can be used on its own for a fistulous tract of small size [4]. It can also be combined with conservative surgical treatment for best results [5].

In our case, the diagnosis was confirmed by CT scan with an injection of contrast medium through the cutaneous fistulous opening. The treatment consisted of the total excision of the fistulous tract, without hysterectomy, with a good clinical outcome.

## Patient consent

Written and informed consent for publication of the case was obtained from the patient.
